# Supplementary material for: Combining deep learning and 3D contrast source inversion in MR‐based electrical properties tomography
Source: NMR Biomed. 2019 Dec 16;35(4):e4211. doi: 10.1002/nbm.4211 (PMC9285035; doi:10.1002/nbm.4211)
Supplement: Supplementary file 1 — Figure S1: MR‐EPT and DL‐EPT reconstructions from 3 T and 7 T B1 + data of the Duke head model. These reconstruction are used as initialization for MR‐CSI and DL‐CSI. Note that a DL‐EPT network at 7 T is not available and these reconstructions are therefore not included. Conductivity (a‐h) and permittivity (i‐p). [file NBM-35-0-s001.pdf]

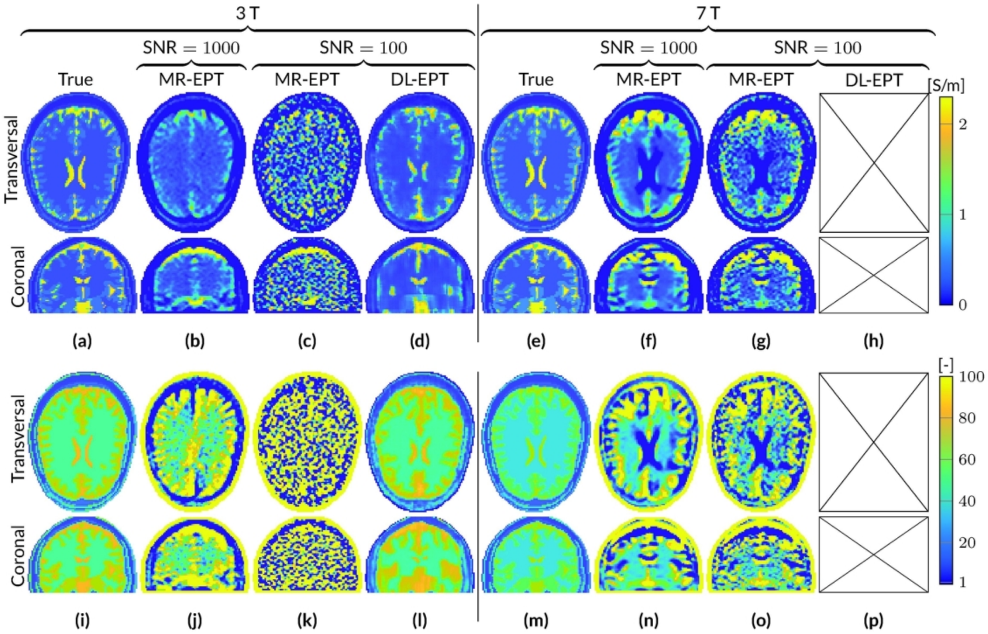

Figure S1: MR-EPT and DL-EPT reconstructions from 3 T and 7 T B_1^+ data of the Duke head model. These reconstruction are used as initialization for MR-CSI and DL-CSI. Note that a DL-EPT network at 7 T is not available and these reconstructions are therefore not included. Conductivity (a-h) and permittivity (i-p).

158x100mm (300 x 300 DPI)
