# Supplementary material for: Combining deep learning and 3D contrast source inversion in MR‐based electrical properties tomography
Source: NMR Biomed. 2019 Dec 16;35(4):e4211. doi: 10.1002/nbm.4211 (PMC9285035; doi:10.1002/nbm.4211)
Supplement: Supplementary file 2 — TABLE S1 The mean and standard deviation of the reconstructed EPs for the segmented regions of white matter (WM), gray matter (GM), and cerebrospinal fluid (CSF) for the Duke head model for two noise levels at 3 and 7 T. The difference in averages between the different SNR cases in MR‐EPT (and thus MR‐CSI) is due to the applied minimum and maximum constraint [file NBM-35-0-s002.docx]

# Supplementary material

**TABLE S1** The mean and standard deviation of the reconstructed EPs for the segmented regions white matter (WM), gray matter (GM) and cerebrospinal fluid (CSF) for the Duke head model for two noise levels at 3 T and 7 T. The difference in averages between the different SNR cases in MR-EPT (and thus MR-CSI) is due to the applied minimum and maximum constraint.

|  |  | Conductivity | | | | | |
| --- | --- | --- | --- | --- | --- | --- | --- |
|  |  | 3 T | | | 7 T | | |
|  |  | WM | GM | CSF | WM | GM | CSF |
|  | True | 0.34 | 0.59 | 2.14 | 0.41 | 0.69 | 2.22 |
| SNR 1000 | MR-EPT | 0.49±0.29 | 0.70±0.52 | 0.72±0.79 | 0.65±0.54 | 0.86±0.70 | 0.79±0.86 |
|  | H-CSI | 0.44±0.16 | 0.57±0.16 | 0.92±0.29 | 0.50±0.16 | 0.72±0.24 | 1.37±0.46 |
|  | MR-CSI | 0.41±0.30 | 0.69±0.54 | 0.89±0.78 | 0.49±0.40 | 0.67±0.55 | 0.93±0.71 |
|  | DL-CSI | 0.41±0.11 | 0.64±0.36 | 1.46±0.55 | 0.46±0.18 | 0.75±0.30 | 1.68±0.51 |
| SNR 100 | MR-EPT | 0.66±0.69 | 0.82±0.82 | 0.78±0.90 | 0.67±0.64 | 0.87±0.75 | 0.81±0.89 |
|  | DL-EPT | 0.43±0.25 | 0.70±0.41 | 1.54±0.61 | - | - | - |
|  | H-CSI | 0.44±0.16 | 0.57±0.16 | 0.90±0.30 | 0.51±0.17 | 0.72±0.25 | 1.32±0.46 |
|  | MR-CSI | 0.64±0.65 | 0.75±0.77 | 0.87±0.89 | 0.51±0.50 | 0.65±0.60 | 0.94±0.73 |
|  | DL-CSI | 0.41±0.23 | 0.64±0.36 | 1.46±0.56 | 0.46±0.19 | 0.75±0.30 | 1.68±0.51 |
|  |  |  |  |  |  |  |  |
|  |  | Permittivity | | | | | |
|  |  | 3 T | | | 7 T | | |
|  |  | WM | GM | CSF | WM | GM | CSF |
|  | True | 52.53 | 73.52 | 84.04 | 43.78 | 60.02 | 70.73 |
| SNR 1000 | MR-EPT | 62.52±22.80 | 70.49±32.93 | 56.40±43.13 | 47.46±25.27 | 53.88±33.05 | 62.36±40.07 |
|  | H-CSI | 61.04±11.83 | 70.14±12.79 | 81.37±13.89 | 46.60±10.07 | 53.20±13.15 | 68.28±18.34 |
|  | MR-CSI | 53.33±28.45 | 66.16±35.34 | 60.65±40.59 | 37.33±24.24 | 40.93±30.71 | 53.80±36.14 |
|  | DL-CSI | 54.83±11.68 | 68.39±12.57 | 79.36±14.96 | 49.33±8.33 | 61.75±11.16 | 69.87±14.07 |
| SNR 100 | MR-EPT | 55.64±43.86 | 60.71±43.92 | 54.40±45.69 | 47.60±30.55 | 53.90±35.27 | 62.00±40.71 |
|  | DL-EPT | 58.06±9.06 | 71.10±8.68 | 80.40±6.53 | - | - | - |
|  | H-CSI | 60.28±12.10 | 69.80±13.25 | 80.60±14.30 | 47.02±10.93 | 52.42±13.88 | 67.96±19.04 |
|  | MR-CSI | 47.85±41.79 | 46.01±42.58 | 50.65±43.44 | 35.97±29.49 | 37.84±32.74 | 50.72±37.35 |
|  | DL-CSI | 54.72±13.70 | 68.19±14.53 | 79.00±16.39 | 49.27±9.01 | 61.75±11.67 | 69.81±14.45 |
